# Crystal structure, spectroscopic characterization and Hirshfeld surface analysis of *trans*-di­aqua­[2,5-bis­(pyridin-4-yl)-1,3,4-oxa­diazole]di­thio­cyanato­nickel(II)

**DOI:** 10.1107/S2056989019008727

**Published:** 2019-06-21

**Authors:** Ferdaousse Rhoufal, Fouad Bentiss, Salaheddine Guesmi, El Mostafa Ketatni, Mohamed Saadi, Lahcen El Ammari

**Affiliations:** a Laboratoire de Chimie de Coordination et d’Analytique, Faculté des Sciences, Université Chouaib Doukkali, BP 20, M-24000 El Jadida, Morocco; b Laboratoire de Catalyse et de Corrosion de Matériaux (LCCM), Faculté des Sciences, Université Chouaib Doukkali, BP 20, M-24000 El Jadida, Morocco; c Laboratory of Organic and Analytical Chemistry, University Sultan Moulay Slimane, Faculty of Science and Technology, PO Box 523, Beni-Mellal, Morocco; d Laboratoire de Chimie Appliquée des Matériaux, Centre des Sciences des Matériaux, Faculty of Sciences, Mohammed V University in Rabat, Avenue Ibn Batouta, BP 1014, Rabat, Morocco

**Keywords:** coordination complex, crystal structure, 1,3,4-oxa­diazole, thio­cyanate, spectroscopy, hydrogen bonds, Hirshfeld analysis

## Abstract

The reaction of 2,5-bis­(pyridin-4-yl)-1,3,4-oxa­diazole (4-pox) and thio­cyanate ions, used as co-ligand with nickel salt NiCl_2_·6H_2_O, produced the title complex, [Ni(NCS)_2_(C_12_H_8_N_4_O)_2_(H_2_O)_2_]. The Ni^II^ atom is located on an inversion centre and is octa­hedrally coordinated by four N atoms from two ligands and two pseudohalide ions, forming the equatorial plane. The axial positions are occupied by two O atoms of coordinated water mol­ecules. In the crystal, the mol­ecules are linked into a three-dimensional network through strong O—H⋯N hydrogen bonds. Hirshfeld surface analysis was used to investigate the inter­molecular inter­actions in the crystal packing.

## Abstract 2

The Ni^II^ atom in the mononuclear title complex has an almost regular octa­hedral N_4_O_2_ coordination geometry. In the crystal, the complex mol­ecules are linked in a three-dimensional network through strong O—H⋯N hydrogen bonds.

## Chemical context   

Bi- or multidentate bridging heterocyclic ligands, in particular thia­diazole and oxa­diazole derivatives, have been used to bind metal ions, thus generating mono- (Guo *et al.*, 2003 ▸), bi- (Mahmoudi & Morsali, 2007 ▸) or multidimensional (Du *et al.*, 2004*a* ▸; Du *et al.*, 2010 ▸; Li *et al.*, 2010*a* ▸) coordination complexes as well as metal–organic framework (MOF) type coordination polymers with potentially inter­esting magnetic (Li *et al.*, 2010*b* ▸; Laachir *et al.*, 2016 ▸; Liu *et al.*, 2003 ▸) and biological (Zine *et al.*, 2017 ▸; Smaili *et al.*, 2017 ▸; Baba Ahmed *et al.*, 2015 ▸; Barboiu *et al.*, 1996 ▸) properties. Employing angular dipyridyl donor ligands 2,5-bis­(pyridin-4-yl)-1,3,4-thia­diazole and 2,5-bis­(pyridin-4-yl)-1,3,4-oxa­diazole (4-pox) with metal salts has allowed the synthesis of transition-metal complexes with different topologies. The counter-anions (PF_6_^−^, ClO_4_^−^, NO_3_^−^, SCN^−^) seem to play an essential role in the architecture of the products obtained, particularly in the case of polymeric compounds (Du, Lam *et al.*, 2004*b* ▸; Huang *et al.*, 2004 ▸; Mahmoudi & Morsali, 2007 ▸). With the thio­cyanate ion (SCN^−^), mononuclear complexes of formula [*M*(4-pox)_2_(NCS)_2_(H_2_O)_2_] have been synthesized; they exhibit an octa­hedral geometry around the metal site with pseudohalide and organic ligands in mutually *trans* positions (Du *et al.*, 2002*b* ▸, Fang *et al.*, 2002 ▸; Du & Zhao, 2004 ▸). Herein we report the synthesis, structural characterizations and Hirshfeld surface analysis of the title complex.
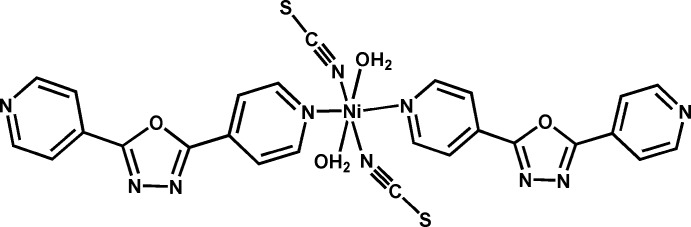


## Structural commentary   

**Figure 1:**
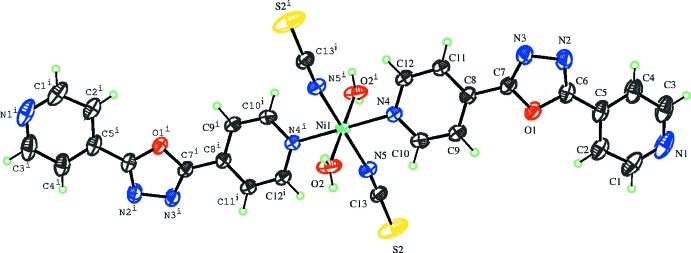
The mol­ecular structure of the title compound with displacement ellipsoids drawn at the 50% probability level. Symmetry code: (i) −*x* + 2, −*y* + 1, −*z* + 1.

In the mol­ecule of the title compound, the nickel(II) cation is located on an inversion centre and shows an almost regular octa­hedral coordination geometry (Fig. 1 ▸). The Ni1 atom is connected to pairs of water mol­ecules and thio­cyanate anions, with Ni1—O2 and Ni1—N5 distances of 2.0748 (18) and 2.0316 (18) Å, respectively. The two remaining, symmetry-related bonds are slightly longer [Ni1—N4 = 2.1327 (17) Å], which leads to a slightly elongated octa­hedral coordination environment. The oxa­diazole ring subtends dihedral angles of 27.86 (13) and 12.74 (14)°, respectively, with the Ni-bound (N4/C8–C12) and outer (N1/C1–C5) pyridine rings while the pyridine rings subtend a dihedral angle of 28.02 (13)°.

## Supra­molecular features   

**Figure 2:**
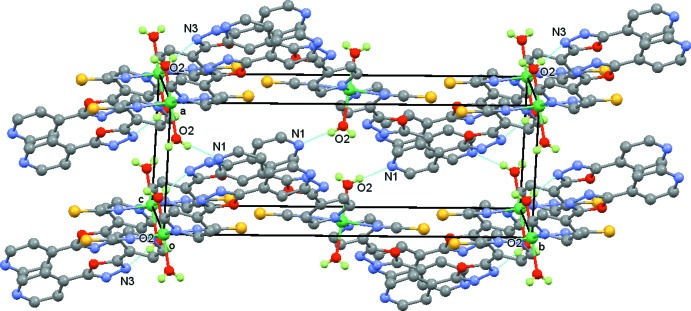
Packing diagram of the title compound viewed approximately along the *c* axis. Hydrogen bonds (Table 1 ▸) are shown as dotted lines.

In the crystal, the mol­ecules are linked through strong O—H⋯N hydrogen bonds (Table 1 ▸), forming a three-dimensional network (Fig. 2 ▸). Weak π–π stacking inter­actions [centroid-to-centroid distance = 3.9749 (12) Å; symmetry operation 2 − *x*, 1 − *y*, 2 − *z*] are also observed between pyridine rings (N4/C8–C12) coordinated to adjacent metal centres.

## Hirshfeld surface analysis   

**Figure 3:**
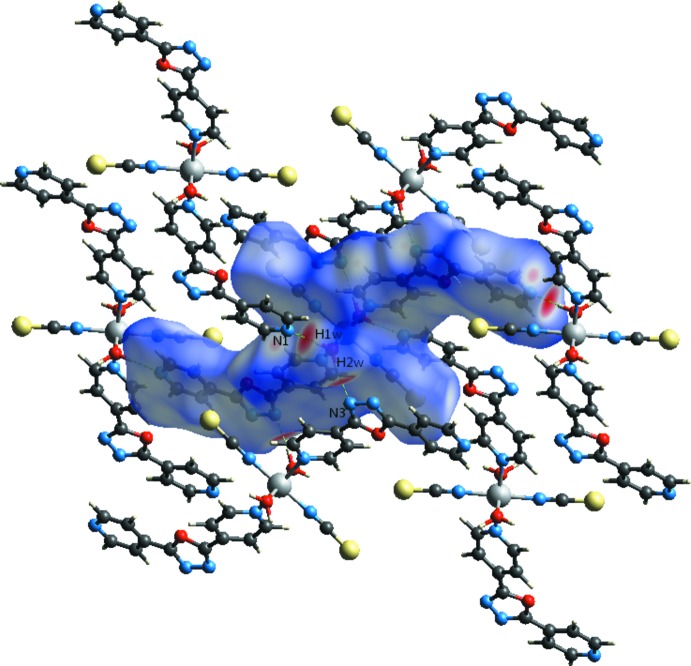
A view of the Hirshfeld surface of the title compound mapped over *d*_norm_, showing the strong O—H⋯N hydrogen bonds (Table 1 ▸; dashed lines).

In order to visualize the role of weak inter­molecular contacts, a Hirshfeld surface (HS) analysis (Spackman & Jayatilaka, 2009 ▸) was carried out and the associated two-dimensional fingerprint plots (McKinnon *et al.*, 2007 ▸) generated using *CrystalExplorer17.5* (Turner *et al.*, 2017 ▸). The three dimensional *d*_norm_ surface of the title compound using a standard surface resolution with a fixed colour scale of −0.6661 to 1.4210 a.u. is shown in Fig. 3 ▸. The darkest red spots on this surface correspond to the O—H⋯N hydrogen bonds resulting from the inter­action between the coordinated water mol­ecules and N atoms of the pyridine and oxa­diazole rings.

**Figure 4:**
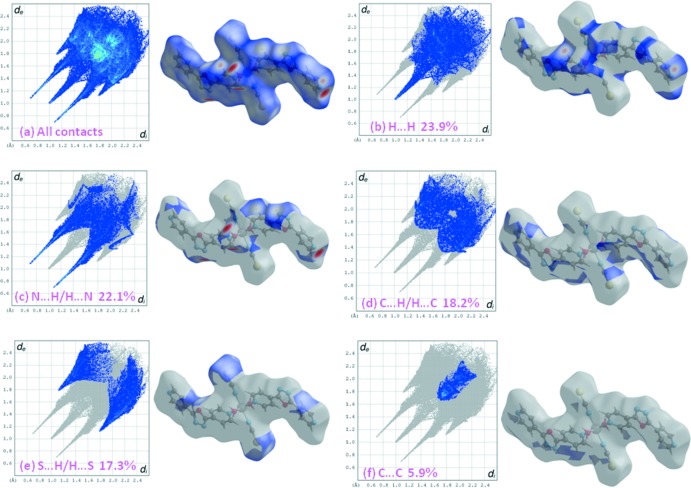
The overall two-dimensional fingerprint plot for the title compound (*a*) and those delineated into (*b*) H⋯H (23.9%), (*c*) N⋯H/H⋯N (22.1%), (*d*) C⋯H/H⋯C (18.2%), (*e*) S⋯H/H⋯S (17.3%) and (*f*) C⋯C (5.9%) contacts.

**Figure 5:**
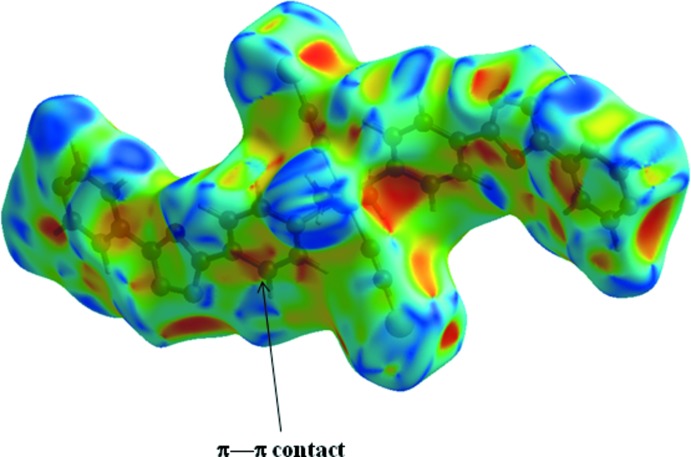
Hirshfeld surface of the title complex plotted over shape-index.

The fingerprint plots in Fig. 4 ▸, for all inter­actions in the title compound, and those delineated into H⋯H, N⋯H/H⋯N, C⋯H/H⋯C, S⋯H/H⋯S and C⋯C contacts, exhibit the characteristic pseudo-symmetric wings in the *d*_e_ and *d*_i_ diagonal axes. The percentage contributions to the overall Hirshfeld surface are given in Table 2 ▸. The H⋯N/N⋯H contacts arising from inter­molecular O—H⋯N hydrogen bonding make a 22.1% contribution to the Hirshfeld surface and are represented by a pair of sharp spikes in the region *d*_e_ + *d*_i_ ≃1.8 Å. In the absence of C—H⋯π inter­actions, the wings in the fingerprint plot delineated into C⋯H/H⋯C contacts (18.2% contribution, Fig. 4 ▸ *d*) also have a nearly symmetrical distribution of points, with thick edges at *d*_e_ + *d*_i_ ≃ 3.1 Å. The H⋯H contacts (23.9% contribution, Fig. 4 ▸ *b*) appear in the central region of the fingerprint plot with *d*_e_ = *d*_i_ ≃ 1.0 Å. The S⋯H/H⋯S contacts (17.3% contribution, Fig. 4 ▸ *e*) indicate that the inter­atomic separations are greater than the sum of the van der Waals radii, suggesting they have a limited influence on the mol­ecular packing. The C⋯C contacts (5.9% contribution, Fig. 4 ▸ *f*) are a measure of the π–π stacking inter­actions and have an arrow-shaped distribution of points with the tip at *d*_e_ = *d*_i_ ≃ 1.7 Å. π–π Inter­actions are indicated by adjacent red and blue triangles in the surface mapped over shape-index (Fig. 5 ▸).

## Spectroscopic characterizations   

FTIR spectra were recorded on a SHIMADZU FT–IR 8400S spectrometer with a Smart iTR attachment and diamond-attenuated total reflectance (ATR) crystal in the range 500–4000 cm^−1^. UV–visible absorption spectra were recorded in the range 200–800 nm using a SHIMADZU 2450 spectrophotometer. The complex concentration used for UV–visible measurements was 10^−4^ *M* in methanol solvent.

**Figure 6:**
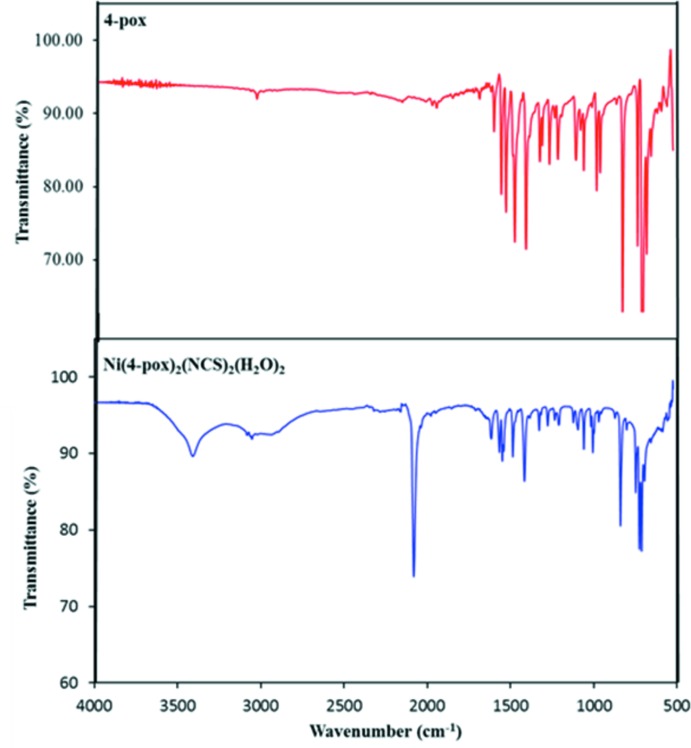
Infrared spectra of the 4-pox ligand and the title complex in the 500–4000 cm^−1^ range.

**Figure 7:**
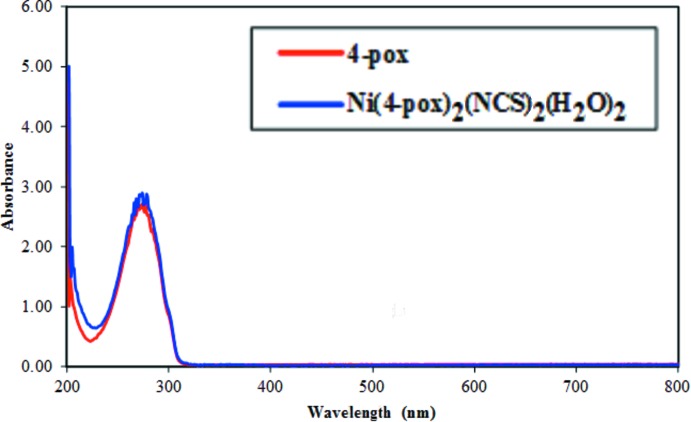
Electronic spectra of the 4-pox ligand and the title complex in methanol (10^−4^ *M*).

The IR spectrum of the title complex (Fig. 6 ▸) is analogous to that of the 4-pox ligand, except for the presence of a wide band of low intensity around 3409 cm^−1^ in addition to another sharp and strong band at 2083 cm^−1^, attributable to the water mol­ecules [υ(OH); Du *et al.*, 2002*a* ▸; Du *et al.*, 2004*a* ▸] and thio­cyanate ions [υ(CN; Du & Zhao, 2004 ▸; Fang *et al.*, 2002 ▸], respectively. A comparison of the spectrum with that of 4-pox, which is characterized by its main absorption bands, 3055–3084, 1618, 1569 and 1551–1418 cm^−1^, resulting from the C—H, C=N (oxa­diazole), C=N (pyridine) and C=C (pyridine) bonds, respectively (Table 3 ▸; Jha *et al.*, 2010 ▸; Formagio *et al.*, 2008 ▸), indicates the presence of 4-pox in the complexes as well as water mol­ecules and thio­cyanate anions in the isolated product, as evidenced by the XRD study. The UV–vis spectrum of the title complex in methanol (Fig. 7 ▸) displays an intense band at 274 nm that is essentially attributable to intra­ligand π–π* electronic transitions in a conjugate system (Mahmoudi & Morsali, 2007 ▸; Kudelko *et al.*, 2015 ▸). The free 4-pox ligand also shows the same band at the same position, indicating that the ligand structure has undergone very few changes upon coordination to the metal.

## Database survey   

A search of the Cambridge Structural Database (CSD, Version 5.40, update of May 2019; Groom *et al.*, 2016 ▸) for six-coordinated metal complexes of 4-pox resulted in 48 hits. The structure of the title compound is similar to those of the related complexes [*M*(4-pox)_2_(NCS)_2_(H_2_O)_2_] where *M* = Cd^II^ (Du *et al.*, 2002*b* ▸), Mn^II^ or Co^II^ (Fang *et al.*, 2002 ▸) or Fe^II^ (Du & Zhao, 2004 ▸). In all cases, an octa­hedral geometry around the metal site with pseudohalide and organic ligands in mutually *trans* positions was observed.

## Synthesis and crystallization   

The 2,5-bis­(4-pyridin-4-yl)-1,3,4-oxa­diazole (4-pox) ligand was synthesized as described previously (Bentiss & Lagrenée, 1999 ▸). To a methano­lic solution (20 ml) of 4-pox (0.2 mmol, 45 mg) under magnetic stirring at room temperature were added successively aqueous solutions (each 5 ml) of KSCN (0.2 mmol, 20 mg) and NiCl_2_·6H_2_O (0.1 mmol, 24 mg). After 10 min of reaction, the precipitate obtained was filtered and washed with distilled water and dissolved in 15 ml of DMF. After one month of slow evaporation of the solvent, the obtained green single crystals were washed with water and dried under vacuum (80%). These crystals were used as isolated for single crystal X-ray analysis. Analysis calculated for C_26_H_20_N_10_NiS_2_O_4_. C, 47.36; H, 3.06; N, 21.24; S, 9.73; found: C, 47.51; H, 3.13; N, 21.29; S, 9.59. IR–ATR (cm^−1^): 3055 (*w*), 1618 (*m*), 1569 (*m*), 1551 (*m*), 1488 (*m*), 1418 (*m*), 1330 (*w*), 1279 (*w*), 1237 (*w*), 1213 (*w*), 1125 (*w*), 1097 (*w*), 1062 (*m*), 1019 (*w*), 1008 (*m*), 972 (*w*), 842 (*s*), 750 (*m*), 728 (*s*), 715 (*s*), 697 (*m*). UV–vis [*λ*_max_, nm (*∊*_max_, *M* ^−1^ cm^−1^)]: 274 (28920).

## Refinement   

Crystal data, data collection and structure refinement details are summarized in Table 4 ▸. The water H atoms were initially located in a difference-Fourier map and refined with O—H distance restraints of 0.78 Å and with *U*_iso_(H) set to 1.5 *U*_eq_(O). All other H atoms were located in a difference-Fourier map and refined as riding, with C—H = 0.93 Å and with *U*_iso_(H) = 1.2*U*_eq_(C).

## Supplementary Material

Crystal structure: contains datablock(s) I. DOI: 10.1107/S2056989019008727/rz5259sup1.cif

Structure factors: contains datablock(s) I. DOI: 10.1107/S2056989019008727/rz5259Isup2.hkl

CCDC reference: 1911306

Additional supporting information:  crystallographic information; 3D view; checkCIF report

## Figures and Tables

**Table 1:** Hydrogen-bond geometry (Å, °)

*D*—H⋯*A*	*D*—H	H⋯*A*	*D*⋯*A*	*D*—H⋯*A*
O2—H1*W*⋯N1^i^	0.77	1.98	2.747 (2)	172
O2—H2*W*⋯N3^ii^	0.78	2.14	2.918 (3)	173

Symmetry codes: (i) 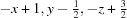; (ii) 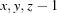.

**Table 2:** Percentage contributions of inter­molecular inter­actions to the Hirshfeld surface in [Ni(4-pox)_2_(NCS)_2_(H_2_O)_2_]

Contact type	Percentage contribution
H⋯H	23.9
N⋯H/H⋯N	22.1
C⋯H/H⋯C	18.2
S⋯H/H⋯S	17.3
C⋯C	5.9
C⋯N/N⋯C	3.7
C⋯O/O⋯C	2.9
C⋯S/S⋯C	2.5
S⋯O/O⋯S	1.4
O⋯H/H⋯O	0.7
N⋯O/O⋯N	0.7
N⋯N	0.5

**Table 3:** IR data (cm^−1^) for the 4-pox ligand and the title complex [Ni(4-pox)_2_(NCS)_2_(H_2_O)_2_]

Bond	4-pox	[Ni(4-pox)_2_(NCS)_2_(H_2_O)_2_]
*C*=*C*(pyridine)	1535–1414 (*m*)	1551–1418 (*m*)
*C*=*N*(pyridine)	1563 (*m*)	1569 (*m*)
*C*=*N*(oxa­diazole)	1608 (*m*)	1618 (*m*)
*C*=*N*(thio­cyanate)	–	2083 (*s*)
C—H	3040 (*w*)	3055 (*w*), 3084 (*w*)
O—H	–	3409 (*m*)

*w* = weak, *m* = medium, *s* = strong.

**Table 4:** Experimental details

Crystal data	
Chemical formula	[Ni(NCS)_2_(C_12_H_8_N_4_O)_2_(H_2_O)_2_]
*M*_r_	659.35
Crystal system, space group	Monoclinic, *P*2_1_/*c*
Temperature (K)	296
*a*, *b*, *c* (Å)	8.5395 (7), 20.7595 (15), 8.6686 (6)
β (°)	108.908 (3)
*V* (Å^3^)	1453.81 (19)
*Z*	2
Radiation type	Mo *K*α
μ (mm^−1^)	0.86
Crystal size (mm)	0.36 × 0.27 × 0.20

Data collection	
Diffractometer	Bruker D8 VENTURE Super DUO
Absorption correction	Multi-scan (*SADABS*; Krause *et al.*, 2015 ▸)
*T*_min_, *T*_max_	0.638, 0.746
No. of measured, independent and observed [*I* > 2σ(*I*)] reflections	19935, 4425, 3100
*R*_int_	0.041
(sin θ/λ)_max_ (Å^−1^)	0.714

Refinement	
*R*[*F* ^2^ > 2σ(*F* ^2^)], *wR*(*F* ^2^), *S*	0.045, 0.123, 1.05
No. of reflections	4425
No. of parameters	197
H-atom treatment	H-atom parameters constrained
Δρ_max_, Δρ_min_ (e Å^−3^)	0.69, −0.76

Computer programs: *APEX3* and *SAINT* (Bruker, 2016 ▸), *SHELXT* (Sheldrick, 2015*a* ▸), *SHELXL2018* (Sheldrick, 2015*b* ▸), *ORTEP-3 for Windows* (Farrugia, 2012 ▸), *DIAMOND* (Brandenburg, 2006 ▸) and *publCIF* (Westrip, 2010 ▸).

## supplementary crystallographic information

### Crystal data

**Table:**

[Ni(NCS)_2_(C_12_H_8_N_4_O)_2_(H_2_O)_2_]	*F*(000) = 676
*M**_r_* = 659.35	*D*_x_ = 1.506 Mg m^−^^3^
Monoclinic, *P*2_1_/*c*	Mo *K*α radiation, λ = 0.71073 Å
*a* = 8.5395 (7) Å	Cell parameters from 4425 reflections
*b* = 20.7595 (15) Å	θ = 2.5–30.5°
*c* = 8.6686 (6) Å	µ = 0.86 mm^−^^1^
β = 108.908 (3)°	*T* = 296 K
*V* = 1453.81 (19) Å^3^	Block, green
*Z* = 2	0.36 × 0.27 × 0.20 mm

### Data collection

**Table:**

Bruker D8 VENTURE Super DUO diffractometer	4425 independent reflections
Radiation source: INCOATEC IµS micro-focus source	3100 reflections with *I* > 2σ(*I*)
HELIOS mirror optics monochromator	*R*_int_ = 0.041
Detector resolution: 10.4167 pixels mm^-1^	θ_max_ = 30.5°, θ_min_ = 2.5°
φ and ω scans	*h* = −11→12
Absorption correction: multi-scan (SADABS; Krause *et al.*, 2015)	*k* = −29→29
*T*_min_ = 0.638, *T*_max_ = 0.746	*l* = −12→12
19935 measured reflections	

### Refinement

**Table:**

Refinement on *F*^2^	Hydrogen site location: mixed
Least-squares matrix: full	H-atom parameters constrained
*R*[*F*^2^ > 2σ(*F*^2^)] = 0.045	*w* = 1/[σ^2^(*F*_o_^2^) + (0.0514*P*)^2^ + 0.6622*P*] where *P* = (*F*_o_^2^ + 2*F*_c_^2^)/3
*wR*(*F*^2^) = 0.123	(Δ/σ)_max_ < 0.001
*S* = 1.05	Δρ_max_ = 0.69 e Å^−^^3^
4425 reflections	Δρ_min_ = −0.76 e Å^−^^3^
197 parameters	Extinction correction: SHELXL2018 (Sheldrick, 2015b), Fc^*^=kFc[1+0.001xFc^2^λ^3^/sin(2θ)]^-1/4^
0 restraints	Extinction coefficient: 0.008 (2)

### Special details

**Table:**

Geometry. All esds (except the esd in the dihedral angle between two l.s. planes) are estimated using the full covariance matrix. The cell esds are taken into account individually in the estimation of esds in distances, angles and torsion angles; correlations between esds in cell parameters are only used when they are defined by crystal symmetry. An approximate (isotropic) treatment of cell esds is used for estimating esds involving l.s. planes.

### Fractional atomic coordinates and isotropic or equivalent isotropic displacement parameters (Å^2^)

**Table:**

	*x*	*y*	*z*	*U*_iso_*/*U*_eq_	
C1	0.5151 (4)	0.85637 (12)	1.0739 (3)	0.0652 (8)	
H1	0.560714	0.891631	1.037771	0.078*	
C2	0.5764 (4)	0.79567 (10)	1.0599 (3)	0.0538 (6)	
H2	0.662391	0.790140	1.017334	0.065*	
C3	0.3276 (4)	0.81602 (14)	1.1842 (4)	0.0680 (8)	
H3	0.242154	0.822966	1.226905	0.082*	
C4	0.3778 (3)	0.75337 (12)	1.1741 (3)	0.0549 (6)	
H4	0.327416	0.719031	1.208419	0.066*	
C5	0.5052 (3)	0.74335 (10)	1.1116 (3)	0.0446 (5)	
C6	0.5599 (3)	0.67774 (9)	1.0969 (3)	0.0411 (5)	
C7	0.6815 (3)	0.60294 (9)	1.0151 (2)	0.0370 (4)	
C8	0.7763 (3)	0.57441 (9)	0.9201 (3)	0.0375 (4)	
C9	0.8891 (3)	0.61127 (10)	0.8754 (3)	0.0484 (6)	
H9	0.916670	0.652504	0.917307	0.058*	
C10	0.9594 (3)	0.58558 (10)	0.7675 (3)	0.0516 (6)	
H10	1.035155	0.610562	0.737470	0.062*	
C11	0.7456 (3)	0.51207 (9)	0.8619 (3)	0.0449 (5)	
H11	0.674855	0.485395	0.894781	0.054*	
C12	0.8219 (3)	0.49036 (9)	0.7543 (3)	0.0472 (6)	
H12	0.800741	0.448454	0.715141	0.057*	
C13	0.9838 (3)	0.64445 (9)	0.3751 (3)	0.0399 (5)	
N1	0.3940 (3)	0.86677 (11)	1.1365 (3)	0.0707 (7)	
N2	0.5248 (3)	0.62564 (9)	1.1590 (3)	0.0516 (5)	
N3	0.6058 (3)	0.57608 (8)	1.1044 (2)	0.0478 (5)	
N4	0.9250 (3)	0.52657 (8)	0.7032 (2)	0.0454 (5)	
N5	0.9805 (3)	0.59299 (8)	0.4230 (3)	0.0478 (5)	
O1	0.65795 (19)	0.66746 (6)	1.00419 (17)	0.0398 (3)	
O2	0.7528 (2)	0.48479 (7)	0.3675 (2)	0.0575 (5)	
H1W	0.703394	0.453575	0.366441	0.086*	
H2W	0.708961	0.506891	0.292497	0.086*	
S2	0.99004 (12)	0.71645 (3)	0.30546 (12)	0.0874 (3)	
Ni1	1.000000	0.500000	0.500000	0.03858 (14)	

### Atomic displacement parameters (Å^2^)

**Table:**

	*U*^11^	*U*^22^	*U*^33^	*U*^12^	*U*^13^	*U*^23^
C1	0.084 (2)	0.0360 (11)	0.0605 (15)	0.0122 (12)	0.0024 (15)	−0.0055 (10)
C2	0.0703 (18)	0.0367 (10)	0.0498 (13)	0.0101 (11)	0.0131 (13)	−0.0037 (9)
C3	0.0566 (18)	0.0648 (17)	0.0704 (18)	0.0273 (14)	0.0038 (15)	−0.0176 (14)
C4	0.0494 (16)	0.0541 (13)	0.0537 (14)	0.0183 (11)	0.0063 (12)	−0.0082 (11)
C5	0.0505 (15)	0.0377 (10)	0.0386 (10)	0.0136 (9)	0.0047 (10)	−0.0048 (8)
C6	0.0467 (13)	0.0361 (10)	0.0389 (10)	0.0080 (9)	0.0116 (10)	−0.0038 (8)
C7	0.0407 (12)	0.0277 (8)	0.0410 (10)	0.0029 (7)	0.0112 (9)	0.0004 (7)
C8	0.0404 (12)	0.0301 (9)	0.0432 (10)	0.0021 (8)	0.0151 (10)	0.0000 (7)
C9	0.0549 (15)	0.0342 (9)	0.0616 (14)	−0.0117 (9)	0.0265 (13)	−0.0127 (9)
C10	0.0563 (16)	0.0392 (10)	0.0696 (15)	−0.0159 (10)	0.0346 (14)	−0.0113 (10)
C11	0.0537 (15)	0.0299 (9)	0.0595 (13)	−0.0042 (8)	0.0301 (12)	−0.0016 (8)
C12	0.0586 (16)	0.0287 (9)	0.0636 (14)	−0.0066 (9)	0.0325 (13)	−0.0062 (8)
C13	0.0403 (13)	0.0310 (9)	0.0456 (11)	−0.0011 (8)	0.0099 (10)	−0.0038 (8)
N1	0.0738 (18)	0.0484 (12)	0.0680 (14)	0.0283 (11)	−0.0071 (13)	−0.0148 (10)
N2	0.0647 (14)	0.0390 (9)	0.0609 (12)	0.0099 (9)	0.0340 (11)	0.0009 (8)
N3	0.0609 (14)	0.0330 (8)	0.0583 (11)	0.0063 (8)	0.0317 (11)	0.0018 (7)
N4	0.0526 (12)	0.0312 (8)	0.0620 (12)	−0.0059 (8)	0.0320 (10)	−0.0062 (8)
N5	0.0527 (13)	0.0311 (8)	0.0629 (12)	−0.0026 (7)	0.0234 (10)	−0.0001 (8)
O1	0.0500 (10)	0.0278 (6)	0.0421 (8)	0.0035 (6)	0.0159 (7)	−0.0007 (5)
O2	0.0481 (11)	0.0392 (8)	0.0786 (12)	−0.0102 (7)	0.0116 (9)	0.0160 (8)
S2	0.0959 (7)	0.0375 (3)	0.0972 (6)	−0.0107 (3)	−0.0123 (5)	0.0245 (3)
Ni1	0.0423 (3)	0.02354 (17)	0.0559 (3)	−0.00321 (14)	0.0241 (2)	−0.00040 (14)

### Geometric parameters (Å, º)

**Table:**

C1—N1	1.332 (4)	C8—C11	1.383 (3)
C1—C2	1.385 (3)	C9—C10	1.372 (3)
C1—H1	0.9300	C9—H9	0.9300
C2—C5	1.388 (3)	C10—N4	1.338 (3)
C2—H2	0.9300	C10—H10	0.9300
C3—N1	1.325 (4)	C11—C12	1.375 (3)
C3—C4	1.381 (3)	C11—H11	0.9300
C3—H3	0.9300	C12—N4	1.338 (3)
C4—C5	1.379 (3)	C12—H12	0.9300
C4—H4	0.9300	C13—N5	1.150 (3)
C5—C6	1.459 (3)	C13—S2	1.619 (2)
C6—N2	1.286 (3)	N2—N3	1.404 (2)
C6—O1	1.352 (3)	N4—Ni1	2.1327 (17)
C7—N3	1.285 (3)	N5—Ni1	2.0316 (18)
C7—O1	1.353 (2)	O2—Ni1	2.0748 (18)
C7—C8	1.456 (3)	O2—H1W	0.7715
C8—C9	1.380 (3)	O2—H2W	0.7846
			
N1—C1—C2	123.3 (3)	C8—C11—H11	120.7
N1—C1—H1	118.4	N4—C12—C11	123.21 (18)
C2—C1—H1	118.4	N4—C12—H12	118.4
C1—C2—C5	117.8 (3)	C11—C12—H12	118.4
C1—C2—H2	121.1	N5—C13—S2	179.0 (2)
C5—C2—H2	121.1	C3—N1—C1	117.8 (2)
N1—C3—C4	123.8 (3)	C6—N2—N3	105.56 (17)
N1—C3—H3	118.1	C7—N3—N2	106.48 (16)
C4—C3—H3	118.1	C12—N4—C10	117.08 (18)
C5—C4—C3	117.9 (3)	C12—N4—Ni1	122.28 (14)
C5—C4—H4	121.1	C10—N4—Ni1	119.80 (14)
C3—C4—H4	121.1	C13—N5—Ni1	172.93 (19)
C4—C5—C2	119.5 (2)	C6—O1—C7	102.77 (15)
C4—C5—C6	119.4 (2)	Ni1—O2—H1W	126.0
C2—C5—C6	121.1 (2)	Ni1—O2—H2W	119.7
N2—C6—O1	112.89 (17)	H1W—O2—H2W	111.7
N2—C6—C5	128.6 (2)	N5^i^—Ni1—N5	180.0
O1—C6—C5	118.47 (19)	N5^i^—Ni1—O2^i^	90.13 (7)
N3—C7—O1	112.30 (17)	N5—Ni1—O2^i^	89.87 (7)
N3—C7—C8	130.17 (17)	N5^i^—Ni1—O2	89.87 (7)
O1—C7—C8	117.41 (17)	N5—Ni1—O2	90.13 (7)
C9—C8—C11	118.93 (19)	O2^i^—Ni1—O2	180.00 (8)
C9—C8—C7	120.01 (17)	N5^i^—Ni1—N4	89.38 (7)
C11—C8—C7	120.85 (19)	N5—Ni1—N4	90.62 (7)
C10—C9—C8	118.35 (19)	O2^i^—Ni1—N4	91.57 (8)
C10—C9—H9	120.8	O2—Ni1—N4	88.43 (8)
C8—C9—H9	120.8	N5^i^—Ni1—N4^i^	90.62 (7)
N4—C10—C9	123.7 (2)	N5—Ni1—N4^i^	89.38 (7)
N4—C10—H10	118.2	O2^i^—Ni1—N4^i^	88.43 (8)
C9—C10—H10	118.2	O2—Ni1—N4^i^	91.57 (8)
C12—C11—C8	118.60 (19)	N4—Ni1—N4^i^	180.0
C12—C11—H11	120.7		

Symmetry code: (i) −*x*+2, −*y*+1, −*z*+1.

### Hydrogen-bond geometry (Å, º)

**Table:**

*D*—H···*A*	*D*—H	H···*A*	*D*···*A*	*D*—H···*A*
O2—H1*W*···N1^ii^	0.77	1.98	2.747 (2)	172
O2—H2*W*···N3^iii^	0.78	2.14	2.918 (3)	173

Symmetry codes: (ii) −*x*+1, *y*−1/2, −*z*+3/2; (iii) *x*, *y*, *z*−1.
